# Power and sample size estimation in microarray studies

**DOI:** 10.1186/1471-2105-11-48

**Published:** 2010-01-25

**Authors:** Wei-Jiun Lin, Huey-Miin Hsueh, James J Chen

**Affiliations:** 1 Division of Personalized Nutrition and Medicine, National Center for Toxicological Research, FDA, Jefferson, AR 72079, USA; 2 Department of Statistics, National Chengchi University, Taipei, Taiwan; 3 Graduate Institute of Biostatistics and Biostatistics Center, China Medical University, Taichung, Taiwan

## Abstract

**Background:**

Before conducting a microarray experiment, one important issue that needs to be determined is the number of arrays required in order to have adequate power to identify differentially expressed genes. This paper discusses some crucial issues in the problem formulation, parameter specifications, and approaches that are commonly proposed for sample size estimation in microarray experiments. Common methods for sample size estimation are formulated as the minimum sample size necessary to achieve a specified sensitivity (proportion of detected truly differentially expressed genes) *on average *at a specified false discovery rate (FDR) level and specified expected proportion (*π*_1_) of the true differentially expression genes in the array. Unfortunately, the probability of detecting the specified sensitivity in such a formulation can be low. We formulate the sample size problem as the number of arrays needed to achieve a specified sensitivity with *95% probability *at the specified significance level. A permutation method using a small pilot dataset to estimate sample size is proposed. This method accounts for correlation and effect size heterogeneity among genes.

**Results:**

A sample size estimate based on the common formulation, to achieve the desired sensitivity on average, can be calculated using a univariate method without taking the correlation among genes into consideration. This formulation of sample size problem is inadequate because the probability of detecting the specified sensitivity can be lower than 50%. On the other hand, the needed sample size calculated by the proposed permutation method will ensure detecting at least the desired sensitivity with 95% probability. The method is shown to perform well for a real example dataset using a small pilot dataset with 4-6 samples per group.

**Conclusions:**

We recommend that the sample size problem should be formulated to detect a specified proportion of differentially expressed genes with 95% probability. This formulation ensures finding the desired proportion of true positives with high probability. The proposed permutation method takes the correlation structure and effect size heterogeneity into consideration and works well using only a small pilot dataset.

## Background

DNA microarray technology provides tools for studying the expression profiles of hundreds or thousands of distinct genes simultaneously. A fundamental goal in microarray studies is to identify a subset of genes that are differentially expressed under experimental conditions of interest. Before conducting a microarray experiment, one important issue that needs to be determined is the number of arrays (replicates) required in order to have adequate power to identify differentially expressed genes.

Many sample size estimation methods have been developed for various Type I error specifications, such as family-wise error rate (FWE) [1-3], false discovery rate (FDR) [4-8], and the number of false positives [7,9]. The sample size for a microarray study is commonly calculated as the number of arrays needed to achieve the specified power *on average *(e.g., [3-6,9,10]). The power, the proportion of truly differentially expressed genes expected to be detected, is known as the sensitivity. With the sample size estimate that is calculated to achieve a specified sensitivity *on average*, the proportion of truly differentially expressed genes detected would frequently be less than the average. Consequently, the sample size calculated tends to give an over-optimistic outcome. Alternatively, Wang and Chen [2], Tsai et al. [7] and Shao and Tseng [8] proposed an alternative formulation: the sample size is calculated to ensure detecting at least the specified sensitivity level with a specified probability. This will be referred to as the (confidence) probability formulation.

When the sample size problem is formulated to achieve the specified sensitivity *on average*, we will show that the needed sample size can be simply calculated using the univariate sample size formula without considering dependency among genes. On the other hand, if the problem is formulated to achieve a specified sensitivity with a specified probability, then it requires estimating a percentile of the distribution of sensitivity. In this case, the dependency among genes needs to be taken into consideration. Tsai et al. [7] presented an approach for controlling the comparison-wise error rate (CWER) under the model of independent or equi-correlated normal distribution with a constant power for all genes. Shao and Tseng [8] proposed a model-free procedure to estimate a general correlation matrix under the normal distribution. They used a dataset of 72 samples to illustrate an estimation of the correlation matrix. However, the size of pilot data is often small, 10 or fewer per group, and the estimated variances of the true positives are often negative (zero) resulting in poor estimate of sample size in our simulation study. Tibshirani [10] proposed a permutation method to estimate the FDR and average sensitivity for assessing a specific sample size. Tibshirani's method requires only a small number of pilot datasets and is completely model-free in the sense that no assumptions on the distribution, effect sizes, and correlations of the test statistics are required. However, the standard deviation estimate (standard error) of a test statistic depends on the sample size. A test statistic from a small sample size will have a larger variation than that from a larger sample size. Since the sample size of a pilot dataset is often small, the cutoff level based on a small pilot dataset often exceeds the true cutoff for needed samples and results in over-estimation of the needed sample size.

This paper presents an overview of the power and parameter specifications, and proposes a permutation procedure for sample size determination under the probability formulation ([2,7,8]). The approach of Tibshirani [10] is improved to attain a more correct permutation distribution by incorporation of an adjustment factor. The proposed method uses a small pilot dataset of 4 to 6 samples per group; the method requires fewer samples than the Tibshirani [10] method when the sample size for the pilot dataset is small relative to the needed sample size. When the sample size for the pilot dataset is large, the proposed method and the Tibshirani [10] method are equivalent.

## Methods

Let *m *denote the number of genes studied in an array of which *m*_0 _and *m*_1 _are the numbers of non-differentially and differentially expressed genes, respectively. Given the significance level *α *(per comparison-wise error rate), the results of *m *tests can be summarized as a 2 × 2 table (Table 1).

**Table 1:** Four possible outcomes when testing m hypotheses.

True State of Nature	Declared significant	Declared Not significant	Total
Null	*V*	*S*	*m*_0_

Alternative	*U*	*T*	*m*_1_

Total	*R*	*m*-*R*	*m*

*V *is the number of true null hypotheses that are falsely rejected;

*U *is the number of true alternative hypotheses that are correctly rejected;

*S *is the number of true null hypotheses that are correctly not rejected;

*T *is the number of alternative hypotheses that incorrectly not rejected;

*R *is the total number of null hypotheses rejected among the *m *tests.

*V*/*m*_0 _is the proportion of genes not differentially expressed that are declared significant, its expectation is the per comparison-wise error rate E(*V*)/*m*_0 _= *α*. *V*/*R *is the proportion of declared significant genes among the total number of significances declared that are, in fact, not differentially expressed. Its expectation is the false discovery rate E(*V*/*R*) = *q*, given *R *> 0. *U*/*m*_1 _is the proportion of truly differentially expressed genes that are correctly declared. In a diagnosis problem, this proportion is often referred as the true positive rate, or the sensitivity. By taking expectation, we have the "average sensitivity", E(*U*)/*m*_1_, denoted by *λ*.

### Sample Size Estimation

In sample size estimation, *m*, *m*_1_, and the (standardized) effect size ***δ ***= (*δ*_1_,..., *δ*_*m*1_) for the differentially expressed genes are pre-specified by the investigator. Estimation of sample size needed to achieve the specified sensitivity *λ*_0_, on average, is straightforward. Since *m*_1 _and *λ*_0 _are pre-specified, given a FDR level q* the corresponding significance level for per comparison-wise error rate *α *can easily be calculated. Setting *α *= [*m*_1 _*λ*_0 _q*]/[*m*_0 _(1-q*)], the FDR will be controlled at q* for sufficiently large *m*_1 _and *m*_0_.

If *δ*_*i *_= *δ*_0 _is constant for all *i*, then the comparison-wise power (1 - *β*) of the univariate test is the same and exactly equal to *λ*_0_. Given *α*, *δ*_0_, and (1 - *β*) = *λ*_0_, the sample size can be based on the univariate sample size calculation and is given as(1)

where *t*_*α *_and *t*_*β *_are the percentiles of a *t*-distribution. If *δ*_*i*_'s are different, then *β*_*i *_= *t*^-1 ^() from Equation (1). The sample size n* can be calculated from the following equation(2)

The needed sample size is *n *= ⌈n*⌉, where ⌈n*⌉ is the smallest integer greater than or equal to n*. Given the sample size *n *as calculated, the outcome of a univariate test on a truly differentially expressed gene can be modeled by a Bernoulli random variable with the success probability at least (1 - *β*_*i*_) since *n *≥ n*. The expected number of true detections is at least *m*_1 _*λ*_0_, regardless of the correlation structure among genes and hence the desired sensitivity can be achieved on average. Most sample size estimation methods are either based on this approach or extensions [3-6,9-11]. However, the sample size calculated under this formulation is inadequate; a simple demonstration under an independent model is shown below.

Given *m*, *π*_1 _(= *m*_1_/*m*), a constant effect size *δ*_*i *_= *δ*_0_, q*, *λ*_0_, and the calculated sample size *n *(based on Equation 1), under the independent model, the total number of truly differentially expressed genes detected *U *is a binomial random variable with success probability (1 - *β*) (≥ *λ*_0 _since *n *≥ n*). The probability *ϕ*_*λ*0 _of identifying at least *λ*_0 _fraction of *m*_1 _differentially expressed genes can be calculated as the sum of the binomial probabilities [2,7]:(3)

The method of using Equation (1) to estimate sample size is referred to as the univariate method. Column 3-5 of Table 2 show the estimated sample size *n*, the average sensitivity *λ *and the probability *ϕ*_*λ*0 _at *λ*_0 _= 0.6, 0.7, 0.8, 0.9. The parameters used in the calculation are: *m *= 2,000, *π*_1 _= 5%, 10%, 20%, *δ*_0 _= 2 and q* = 0.05. It can be seen that the probability *ϕ*_*λ*0 _can be less than 60%. That is, using this formulation to calculate needed arrays may result in that an experiment will have the sensitivity less than the specified *λ*_0 _level with more than 40% probability.

**Table 2:** Average formulation versus 95% probability formulation under the independent model.^a^

		Average formulation: Univariate method			95% probability formulation: Binomial method		
			
*π*_1_	*λ*_0_	*n*^b^	*λ*	*ϕ*_*λ*0_	*n*^c^	*λ*	*ϕ*_*λ*0_
5%	60%	9	0.70	0.985	9	0.70	0.985
	70%	9	0.70	0.576	10	0.81	0.997
	80%	10	0.81	0.681	11	0.88	0.993
	90%	12	0.92	0.866	13	0.95	0.992
10%	60%	8	0.70	0.999	8	0.70	0.999
	70%	8	0.71	0.687	9	0.82	1.000
	80%	9	0.82	0.841	10	0.89	1.000
	90%	11	0.93	0.977	11	0.93	0.977
20%	60%	7	0.72	1.000	7	0.72	1.000
	70%	7	0.74	0.975	7	0.74	0.975
	80%	8	0.85	0.996	8	0.85	0.996
	90%	9	0.91	0.792	10	0.95	1.000

a. Estimated sample size *n*, average sensitivity *λ *and probability *ϕ*_*λ*0 _for the specified sensitivity *λ*_0 _= 60%, 70%, 80%, 90%, under the independent model. The parameters used in the calculation were: *m *= 2,000, *π*_1 _= 5%, 10%, 20%, *δ*_0 _= 2 and q* = 0.05.

b. Sample size *n *is computed by the univariate method from Equation (1) to achieve sensitivity *λ*_0 _on average.

c. Sample size *n *is calculated using Tsai et al. [7] method to ensure the probability *ϕ*_*λ*0 _of detecting at least *λ*_0 _fraction of differentially expressed genes is at least 95%.

Alternatively, Wang and Chen [2] formulated the problem as: the number of arrays needed to achieve the specified sensitivity *λ *_0 _with a probability *ϕ *_*λ*0_. In this formulation both *λ *_0 _and *ϕ *_*λ*0 _need to be specified and not necessarily equal. The *ϕ *_*λ*0 _is set at 95% since it is consistent with the common statistical practice of using the 95% confidence probability. Under this formulation, for specified *λ *_0 _the needed number of arrays is calculated so that the average sensitivity is greater than *λ *_0 _and the 5^th ^percentile, *λ *_5_, of the distribution of the sensitivity *U*/*m*_1 _is greater than *λ*_0_:

In the independent and constant effect size model, Tsai et al. [7] used Equations (1) and (3) to estimate the needed sample size which is referred to as the Binomial method. Columns 6-8 of Table 2 show the estimated sample size *n*, the average sensitivity *λ*, and the probability *ϕ*_*λ*0 _for *λ*_0 _= 0.6, 0.7, 0.8, 0.9. The probabilities in Column 8 are all higher than 95% due to *n *≥ n*. The procedure will ensure detecting the specified proportion of differentially expressed genes with at least 95% probability.

In Table 2, the theoretical results indicate that the two methods give quite close sample size estimates. The difference of the estimates reflects the difference of the two formulations; when *δ*_0 _= 2, the difference is up to 1. For a given sensitivity, the needed sample size increases as the effect size *δ*_0 _decreasing, and the difference of the two formulations in the estimates is larger. We calculated the sample sizes using the same parameters as Table 2 for *δ*_0 _= 1. The sample size differences increase at about four times those of Table 2 (data not shown).

### Permutation Method for Sample Size Estimation

Tibshirani [10] proposed a permutation method to account for both dependency and unequal effect sizes among genes using a pilot dataset for assessing sample size. This method is applied here to estimate the required sample size. Because the sample size of the pilot data is typically smaller than the needed sample size, the null distributions generated from the pilot data have more variations; simply using the null distributions generated from a small pilot dataset can overestimate the needed sample size. A procedure modified from the Tibshirani [10] method with adequate adjustment for sample size estimation is proposed below.

For simplicity, assume an equal sample size in each group, denoted as *n *= *n*_0 _= *n*_1_. Start with some pilot data with at least 4 samples per group, denoted as *n*_0p _and *n*_1p _for the control and treatment group, respectively. For specified *m*, *m*_1_, ***δ ***= (*δ*_1_, ..., *δ*_*m*1_), q*, and *λ*_0_, the algorithm for a two sample *t*-test is described as follows.

Algorithm: Sample Size Estimation (See additional file 1 for a software application)

1. Set *α *= [*m*_1 _*λ*_0_q*]/[*m*_0_(1 - q*)], use the method of Tsai et al. [7] (Column 6 of Table 2) to find the needed sample size as the initial sample size *n*.

2. Compute the adjustment factor *f *= *f*_1 _*f*_2 _where , , and *t*_*df*, *p *_is the *p*^th ^percentile of a *t*-distribution with *df *degrees of freedom.

3. Generate the *b*-th permutation samples.

4. Compute the *t*-statistics and sample standard deviations for the permutation samples for all genes.

5. Multiply each *t*-statistic by the factor *f *and add  to a set of randomly selected *m*_1 _*t*-statistic of differentially expressed genes to generate the permutation *t*-statistics ***s***_*b *_= {***s***_0*b*, _***s***_1*b*_}, where ***s***_0*b *_is the set for the non-differentially expressed genes, and ***s***_1*b *_is the set for the differential expressed genes such that ***s***_0*b *_= *f****t***_0*b *_and ***s***_1*b *_= *f****t***_1*b *_+ , where ***t***_0*b *_and ***t***_1*b *_are the vectors of the *t*-statistic, ***δ ***is a vector of the effect size and  is the vector of the sample standard deviation.

6. Store the permutation statistics ***s***_*b*_.

7. Repeat 3-6 for all possible permutations, *b *= 1, 2, ..., *N*, where N = (*n*_0p_+*n*_1p_) **C***n*_0p_

8. Construct the null distribution by pooling all permutation statistics from the set of non-differentially expressed genes ***s***_0 _= {***s***_01_, ***s***_02_, ..., ***s***_0*N*_}. Find the 100×(*α*/2)^th ^and 100×(1 - *α*/2)^th ^percentiles as the critical values.

9. Compute the number of significances for the true positives *u*_*b *_for each statistic in ***s***_1*b *_for each permutation sample *b *= 1, 2, ..., *N*.

10. Order *u*_1_, *u*_2_, ..., *u*_*N*_, and find the 5^th ^percentile, denoted by u*.

11. Compare u* to *m*_1 _*λ*_0_. If u* ≥ *m*_1_*λ*_0_, stop and report *n *as the sample size estimate; otherwise, increase *n *by 1 and go to 2.

In the proposed algorithm, the permutation *t*-statistics of non-differentially expressed genes from all possible permutations were pooled to estimate the null distribution of the test statistics (Step 8). The number of true positives (*U*) was estimated for each permutation sample (Step 9) since the set of differentially expressed genes in each permutation sample were known. The distribution of the number of true positives *U *and its 5^th ^percentile u* were estimated (Step 10). To reduce the excess variation of the permutation distribution, the proposed method includes the adjustment factor: *f *= *f*_1_*f*_2_. The adjustment factor consists of two scale factors: *f*_1 _and *f*_2_. The first factor, *f*_1_, accounts for differential sample sizes between the pilot study and the planned study and the second scale factor, *f*_2_, uses the maximum likelihood estimate of the *t*-statistic [12]. When the sample size of pilot data is large, both factors *f*_1 _and *f*_2 _converge to 1 and the proposed and the Tibshirani [10] methods are equivalent. (Note that Tibshirani's method was proposed based on the average formulation.) Since the permutation technique is used to estimate the critical value and the distribution of the sensitivity, no assumptions on the distribution of the *t*-statistic and the dependency among the statistics are made. Furthermore, the proposed method does not need to estimate the covariance matrix among all genes which can result in computation difficulty when the sample size of the pilot dataset is small.

## Results

Two simulation analyses were conducted to evaluate the two formulations of sample size estimation described above. The first analysis evaluated the two formulations under the independent and constant effect size model. The theoretical results for the two formulations are shown in Table 2. The simulation analysis provides an empirical validation. The second analysis evaluated the four methods under a correlated model: 1) the univariate method (e.g., Jung [4]); 2) the Shao and Tseng [8] model-free method, 3) the Tibshirani [10] permutation method; and 4) the proposed permutation method. The univariate method is designed for the average formulation, while the three other methods are considered with 95% probability with a use of a pilot dataset. The same model parameters in Table 2 were used in the evaluation. The Type I error rate was based on setting the FDR at q* = 0.05. Note that there are many multiple testing FDR procedures with different strategies. For example, the Storey's FDR procedure [13] involved an estimation of the number of non-differentially expressed genes *m*_0_. However, to minimize the confounding effect brought by the variation in estimating *m*_0_, we simply used the true *m*_0 _in our simulation analysis. Sample sizes were calculated for the given parameter values. The empirical estimates of the FDR, average sensitivity *λ *and the probability *ϕ*_*λ*0 _were then calculated and evaluated. Using the true *m*_0 _provides a direct validation of the proposed procedure with control of the FDR.

The purpose of the first simulation study was to validate the theoretical results of the sample size, sensitivity, and 95% probability for the two methods shown in Table 2 under the independent model. We generated 1,000 simulation samples with sample sizes per group from the Column 3 or Column 6 of Table 2. For the null model, *m*_0 _= *m *× (1 - *π*_1_) genes were generated from the independent standard normal N(0,1); for the alternative model, *m*_1 _= *m *× *π*_1 _genes were generated based on independent normal N(*δ*_0_, 1). For each simulation sample set, the *t*-statistics and the correspondent p-values were computed, and the numbers of false positives and true positives at the FDR level q* = 0.05 were recorded. The empirical estimates of the FDR, average sensitivity *λ *and probability *ϕ*_*λ*0 _were then calculated. The estimate of *ϕ*_*λ*0 _was the proportion of times out of the 1,000 simulations that the number of true positives was not less than *m*_1 _× *λ*_0_.

Table 3 shows the empirical results for the two methods. The empirical FDR appears close to the nominal levels in both approaches. For the univariate method, the empirical average sensitivity *λ*'s are all at or above the desired levels, except for *π*_1 _= 0.05 and *λ*_0 _= 70%. The probability *ϕ*_*λ*0 _is less than 50%, for *π*_1 _= 0.05 and *λ*_0 _= 70%. For the binomial method, the empirical average sensitivities *λ*'s are all greater than the specified levels. Most of probabilities *ϕ*_*λ*0_'s exceed 95% except for *π*_1 _= 0.05, *λ*_0 _= 60%, *π*_1 _= 0.10, *λ*_0 _= 90% and *π*_1 _= 0.20, *λ*_0 _= 70%. The empirical results of Table 3 are generally consistent with the theoretical values shown in Table 2. That is, the sample size calculated using the univariate method generally will achieve the specified sensitivity on average; however, the probability to achieve the specified sensitivity can be lower than 50%.

**Table 3:** The validation of the theoretical results from Table 2.^a^

		Average formulation				95% probability formulation				
			
		Univariate method				Binomial method				Permutation
***π*_1_**	***λ*_0_**	***n*^*b*^**	***q***	***λ***	***ϕ*_*λ*0_**	***n*^*c*^**	***q***	***λ***	***ϕ*_*λ*0_**	***n*^*d*^**

5%	60%	9	0.0505	0.69	0.937	9	0.0505	0.69	0.937	11.3(0.453)
	70%	9	0.0505	0.69	0.497	10	0.0502	0.80	0.983	12.5(0.507)
	80%	10	0.0502	0.80	0.506	11	0.0494	0.87	0.961	14.2(0.485)
	90%	12	0.0492	0.91	0.730	13	0.0484	0.95	0.965	17.1(0.568)
10%	60%	8	0.0490	0.71	0.997	8	0.0490	0.71	0.997	9.8(0.361)
	70%	8	0.0490	0.71	0.589	9	0.0506	0.81	1.000	10.8(0.368)
	80%	9	0.0506	0.81	0.688	10	0.0503	0.88	0.999	12.1(0.291)
	90%	11	0.0497	0.93	0.921	11	0.0497	0.93	0.921	14.6(0.491)
20%	60%	7	0.0498	0.73	1.000	7	0.0498	0.73	1.000	8.0(0.089)
	70%	7	0.0498	0.73	0.901	7	0.0498	0.73	0.901	9.0(0.045)
	80%	8	0.0491	0.84	0.966	8	0.0491	0.84	0.966	10.1(0.224)
	90%	9	0.0501	0.90	0.627	10	0.0497	0.94	0.999	12.2(0.384)

a. Empirical estimates of FDR *q*, average sensitivity *λ*, and probability *ϕ*_*λ*0 _of the univariate method for the average formulation and of the binomial method for the 95% probability formulation. The parameters used in the calculation were: *m *= 2,000, *δ*_0 _= 2, and q* = 0.05.

b. Sample size *n *is computed by the univariate method from Equation (1) to achieve sensitivity *λ*_0 _on average.

c. Sample size *n *is calculated using Tsai et al. [7] method to ensure sensitivity *λ*_0 _with 95% probability.

d. Sample size *n *(standard deviation) is calculated using the proposed permutation method to ensure sensitivity *λ*_0 _with 95% probability with pilot study of group size 4 under the independent model.

For comparison purposes, the mean and standard deviation of the sample size estimates from the proposed permutation method using a pilot dataset of group size 4 are also provided in the last column of Table 3. The pilot data were randomly generated from the normal distribution in each simulation. The proposed method tends to over-estimate the needed sample size by up to five arrays.

The second analysis was to evaluate the four methods, the univariate method (Jung [4]), Shao and Tseng [8], Tibshirani [10], and proposed permutation methods, under a correlated model using the well known colon cancer dataset [14]. The colon cancer dataset [14] consists of 22 normal and 40 colon tumor tissue samples with 2,000 genes. The analysis consisted of two steps. The first step evaluated the sample size estimates obtained by the three 95% probability formulation methods based on a pilot dataset of sample size 4 and 6 per group. The second step compared the sample sizes estimated by the proposed method from the first step with the estimates from the univariate method.

In the first step, 4 samples from the colon dataset were randomly selected without replacement from each group to form a pilot dataset. The algorithm described above was used to estimate the sample size for the proposed method and the Tibshirani [10] method. For example, for *π*_1 _= 5%, q* = 0.05 and *λ*_0 _= 90%, the initial sample size was *n *= 13 (Column 6 of Table 2) and *α *= 0.00249. A constant effect size *δ*_*i *_= *δ*_0 _= 2 was considered. For the proposed permutation method, the initial adjustment factors for *f *were *f*_1 _= 0.6777 and *f*_2 _=  = 1.155, while no adjustment was taken for the Tibshirani [10] method. For the Shao and Tseng [8] model-free method, a correlation matrix of *t*-statistics was estimated by using all possible permutation datasets from the pilot dataset. However, the Shao and Tseng [8] model-free method was found to have computational difficulty in most cases. Details are given later.

The procedure was repeated 1,000 times to select different pilot datasets of size 4 from each group to account for the variation of pilot dataset. The means and standard deviations of the sample size estimates from the Tibshirani [10] and proposed methods were calculated and are shown in Columns 4 and 5 of Table 4. The univariate method is considered as the standard method, and the estimates are listed in Column 3. The needed sample size estimated from either the Tibshirani [10] or the proposed method is greater than that from the univariate method in each case. The difference between the univariate method and the proposed method is less than 5 arrays per group in each case. The mean and standard deviation estimates from the Tibshirani [10] method are much larger than the estimates from the proposed method. The difference increases as *λ*_0 _increases or *π*_1 _decreases. Note that, under the independent model, the sample size and standard deviation estimates from the proposed method are smaller (Table 3).

**Table 4:** Sample size estimates (standard deviations) for the proposed method and the Tibshirani [10] permutation method under a correlated model with effect size 2.^a^

			Pilot study of group size 4		Pilot study of group size 6		Entire data of size 62
					
*π*_1_	*λ*_0_	*n*^b^	*n*^c^	*n*^d^	*n*^c^	*n*^d^	*n*^e^
5%	60%	9	12.2(2.931)	20.2(6.529)	12.7(2.193)	14.9(3.347)	9.5
	70%	9	13.1(2.848)	21.6(6.209)	13.4(2.330)	15.9(3.504)	10.3
	80%	10	14.3(3.017)	23.6(6.399)	14.4(2.335)	17.2(3.547)	11.5
	90%	12	16.3(2.997)	27.1(6.303)	16.1(2.365)	19.5(3.559)	13.7
10%	60%	8	10.9(2.409)	15.7(4.664)	11.5(2.015)	12.5(2.828)	8.1
	70%	8	11.8(2.544)	16.8(4.858)	12.1(2.096)	13.4(2.971)	8.8
	80%	9	13.0(2.601)	18.6(4.809)	13.0(2.033)	14.4(2.852)	9.8
	90%	11	14.7(2.944)	21.5(5.250)	14.6(2.275)	16.4(3.099)	11.8
20%	60%	7	9.8(2.184)	12.2(3.608)	10.3(1.832)	10.4(2.390)	6.7
	70%	7	10.4(2.236)	12.8(3.675)	10.7(1.899)	10.9(2.446)	7.3
	80%	8	11.4(2.414)	14.2(3.709)	11.6(1.995)	11.9(2.506)	8.2
	90%	9	13.1(2.515)	16.5(3.902)	13.0(2.074)	13.6(2.603)	9.9

a. The sample size estimates are based on 1,000 repetitions using the colon tumor data [14] with 4 and 6 samples from each group as pilot dataset. The parameters used in the calculation were: *m *= 2,000, *δ*_0 _= 2 and q* = 0.05.

b. The univariate method.

c. The proposed permutation method

d. The Tibshirani [10] permutation method.

e. The Shao and Tseng [8] model-free method using the entire 62 samples.

The procedure was repeated with 6 samples for the initial pilot dataset. The estimates are shown in Columns 6 and 7. The proposed procedure gives consistent results from the two pilot sample sizes; however, the results from the Tibshirani [10] method differ substantially. The Tibshirani approach does not adequately take the pilot sample size into consideration. When the pilot sample size is much smaller than the needed sample size, the overestimation of the sample size by Tibshirani [10] method becomes severe. As the pilot study size getting closer to the needed sample size, the Tibshirani [10] and the proposed methods will give similar results.

In our simulations, the Algorithm B in Shao and Tseng [8] couldn't successfully produce solutions for the pilot data of group size 4 in all 1,000 replications. When the group size increases to 6, the algorithm works only when *π*_1 _= 20%, *λ*_0 _= 60% and 70%; the mean (standard deviation) of the sample size estimates are 6.4(0.012) and 6.8(0.012), respectively. The estimated values appear too small to be correct. This method does not appear to be applicable for small pilot sample sizes. Using the entire colon cancer dataset [14] of 62 samples, the sample size estimates are shown in Column 8. The estimates generally need one or two more arrays than the univariate methods, but fewer than the proposed method. Since the Tibshirani [10] method gave larger estimates and the Shao and Tseng [8] gave smaller estimates than the proposed method. In the second step of analysis, the univariate method and the proposed method were evaluated.

Comparison of the performance of the two methods is similar to that shown in Table 3. The data were sampled without replacement from the colon cancer dataset, instead of from the normal random variables under the independent model. The sample sizes were based on Column 3 or Column 4 of Table 4. The data were then randomly permuted to remove the difference between two groups, and a common effect size *δ*_0 _= 2 was added to a set of randomly selected *m*_1 _genes in the tumor group. For each re-sampled data set, the permutation test was used to generate a p-value and the numbers of false positives and true positives were computed using q* = 0.05. The number of repetitions to compute the permutation test was 10,000. The empirical estimates of FDR, *λ *and *ϕ*_*λ*0 _were computed. The entire procedure was repeated 1,000 times.

Table 5 shows the empirical estimates of q*, *λ*, and *ϕ*_*λ*0 _for the two methods. Both methods are shown to control the FDR well and achieve the desired sensitivity. Thus the two methods can be expected to have satisfactory performance in practice. However, for the univariate method, the empirical *ϕ*_*λ*0 _estimates are between 55% and 75%, except one at 80%. One would have to take a risk that the sensitivity can fall below the specified level.

**Table 5:** Empirical estimates of FDR, average sensitivity *λ*, and probability *ϕ*_*λ*0 _from the univariate method and the proposed method based on the results of Table 4.

		Average formulation: Univariate method				95% probability formulation: Proposed method			
			
*π*_1_	*λ*_0_	*n*	*q*	*λ*	*ϕ*_*λ*0_	*n*	*q*	*λ*	*ϕ*_*λ*0_
5%	60%	9	0.0412	0.65	0.661	13	0.0431	0.94	0.976
	70%	9	0.0424	0.65	0.558	14	0.0443	0.97	0.984
	80%	10	0.0389	0.76	0.611	15	0.0458	0.99	0.993
	90%	12	0.0460	0.91	0.743	17	0.0426	1.00	0.998
10%	60%	8	0.0427	0.66	0.666	11	0.0474	0.92	0.964
	70%	8	0.0419	0.66	0.585	12	0.0478	0.96	0.973
	80%	9	0.0431	0.78	0.666	13	0.0450	0.98	0.981
	90%	11	0.0466	0.92	0.800	15	0.0475	1.00	0.994
20%	60%	7	0.0433	0.69	0.711	10	0.0447	0.94	0.975
	70%	7	0.0448	0.69	0.634	11	0.0498	0.97	0.987
	80%	8	0.0428	0.81	0.703	12	0.0496	0.99	0.994
	90%	9	0.0442	0.89	0.716	14	0.0488	1.00	1.000

The effect size of *δ*_0 _= 2 (Table 4) was used to validate the proposed permutation method under a correlated model using the colon cancer dataset [14]. In practice, the effect sizes can be much smaller. We calculated the sample sizes using the same parameters as Table 4 with an effect size *δ*_0 _= 1 for two pilot sample sizes 4 and 6. The sample size estimates are shown in Table 6. The proposed procedure gives similar results for the two pilot sample sizes, which are consistent with the results for *δ*_0 _= 2 in Table 4. The difference between the univariate method and the proposed method is about 15 arrays per group. The Tibshirani [10] method would require up to 67 and 35 extra arrays per group for 4 and 6 pilot samples, respectively. The estimates for the Shao and Tseng [8] method could be estimated only when the pilot study size is around or larger then the needed sample size.

**Table 6:** Sample size estimates (standard deviations) for the proposed method and the Tibshirani [10] permutation method under a correlated model with effect size 1.^a^

			Pilot study of group size 4		Pilot study of group size 6		Entire data of size 62
					
*π*_1_	*λ*_0_	*n*^b^	*n*^c^	*n*^d^	*n*^c^	*n*^d^	*n*^e^
5%	60%	26	39.4(11.166)	77.8(22.283)	40.5(8.743)	56.8(12.376)	29.0
	70%	29	43.0(11.659)	84.5(24.442)	43.5(8.913)	61.1(13.570)	31.7
	80%	33	48.7(13.104)	92.2(23.398)	47.8(9.138)	65.4(13.134)	NaN
	90%	40	56.8(13.846)	106.3(25.373)	54.3(9.168)	74.1(13.846)	NaN
10%	60%	23	34.9(9.140)	60.9(18.692)	36.8(8.074)	48.5(12.212)	25.0
	70%	26	38.8(9.821)	66.2(18.993)	40.0(8.408)	52.0(11.819)	27.8
	80%	29	43.3(10.399)	72.5(18.492)	43.3(8.475)	55.8(11.662)	31.4
	90%	35	50.2(10.649)	83.7(20.271)	49.5(8.593)	64.0(12.485)	NaN
20%	60%	19	31.1(9.066)	47.0(14.301)	32.6(7.572)	39.8(9.552)	20.7
	70%	22	34.4(8.740)	50.4(14.156)	35.7(7.816)	42.6(9.570)	23.4
	80%	25	38.6(9.611)	55.5(15.393)	39.0(7.766)	46.6(10.415)	27
	90%	31	44.7(9.655)	63.6(14.919)	44.5(7.999)	52.3(10.313)	32.3

a. The sample size estimates are based on 1,000 repetitions using the colon tumor data [14] with 4 and 6 samples from each group as pilot dataset. The parameters used in the calculation were: *m *= 2,000, *δ*_0 _= 1 and q* = 0.05.

b. The univariate method.

c. The proposed permutation method

d. The Tibshirani [10] permutation method.

e. The Shao and Tseng [8] model-free method using the entire 62 samples.

## Discussion and Conclusions

Determination of the needed sample size before conducting a microarray experiment is an important issue. The sample size problem is commonly formulated as the number of arrays needed to achieve the specified sensitivity *λ *on average. This paper demonstrates that the calculated sample size under this formulation may have the sensitivity *λ *at the specified level on average, but, the probability *ϕ*_*λ *_that the specified sensitivity is achieved can be low (less than 50%) due to the variance in sensitivity distributions. Furthermore, under this formulation this paper shows that the sample size can be calculated by a univariate method, regardless of the correlation structure among the gene expression levels; the procedures to account for correlations, such as Li et al. [6], are not needed (Table 5). These findings agree with the results reported by Jung [4] and Dobbin and Simon [11]. However, this paper provides a theoretical interpretation for this approach.

Under the confidence probability formulation, consideration of the dependency among gene expressions is necessary in estimating the sample size since the percentile of the sensitivity distributions not only depends on the effect size of individual genes but also on their correlations. We propose a permutation method based on the method proposed by Tibshirani [10], but with an inclusion of an adjustment factor and a requirement to achieve a specific sensitivity with 95% probability. The adjustment factor provides more accurate estimates of the power and sample size. Shao and Tseng [8] also formulated the needed sample size in terms of confidence probability. Under the normality assumption, Shao and Tseng [8] proposed algorithms for mild correlations among genes using a preliminary dataset. They showed that their approach worked well for an example dataset of 72 samples. However, using their Algorithm B in our simulation for the colon dataset (the average correlation for the colon dataset is about 0.4), the estimated variance of the true positives can be negative when the preliminary sample size is 4 or 6. Their procedure does not perform well for a small pilot dataset with small sample size. In practice, sample sizes of pilot data are often small. Our simulation studies show that our procedure can work well with 4 to 6 samples per group. However, our procedure seems to over-estimate the needed sample size when the correlations are very small, especially with small effect sizes. In this situation, our simulation results indicate that the factor *f*_2 _may not be necessary (data not shown).

The choice of a particular multiple testing procedure used for data analysis can affect the error rate and power in the sample size estimation. Using a conservative procedure in the data analysis may decrease the "power" of the study; sometimes, the calculated sample size may have sensitivity below the specified level. For example, in this paper the calculation is based on the true number of non-differentially genes *m*_0_. However, if the data analysis uses an overestimated *m*_0 _such as the Benjamini and Hochberg procedure [15], then the power may be below the desired level. An alternative is to use the total number of genes *m *instead of the number of non-differentially genes *m*_0 _to estimate the sample size. This procedure is expected to generate an appropriate sample size to achieve the desired sensitivity with a specified probability, regardless of which multiple testing procedure is used for data analysis.

## Authors' contributions

JJC conceived the study and wrote the manuscript. JJC and WJL developed the methodology and proved theoretical results. WJL implemented the algorithms. HMH improved the concepts of the average and 95% confidence probability formulations. JJC, HMH and WJL performed the analysis. All authors read and approved the final manuscript.

## Supplementary Material

Additional file 1 **The software for the algorithm of the proposed method**. It provides software and an example for the algorithm of the proposed method. Click here for file
